# Cesarean Section or Vaginal Delivery to Prevent Possible Vertical Transmission From a Pregnant Mother Confirmed With COVID-19 to a Neonate: A Systematic Review

**DOI:** 10.3389/fmed.2021.634949

**Published:** 2021-02-17

**Authors:** Jianghui Cai, Mi Tang, Yu Gao, Hongxi Zhang, Yanfeng Yang, Dan Zhang, Han Wang, Hua Liang, Rui Zhang, Bo Wu

**Affiliations:** ^1^Department of Pharmacy, Chengdu Women's and Children's Central Hospital, School of Medicine, University of Electronic Science and Technology of China, Chengdu, China; ^2^Office of Good Clinical Practice, Chengdu Women's and Children's Central Hospital, School of Medicine, University of Electronic Science and Technology of China, Chengdu, China; ^3^Department of Pediatric Cardiology, Chengdu Women's and Children's Central Hospital, School of Medicine, University of Electronic Science and Technology of China, Chengdu, China

**Keywords:** coronavirus disease 2019, COVID-19, SARS-CoV-2, pregnancy, mode of delivery, vertical transmission

## Abstract

**Background:** The impact of delivery mode on the infection rates of Coronavirus disease 2019 (COVID-19) in the newborn remains unknown. We aimed to summarize the existing literature on COVID-19 infection during pregnancy to evaluate which mode of delivery is better for preventing possible vertical transmission from a pregnant mother confirmed with COVID-19 to a neonate.

**Methods:** We performed a comprehensive literature search of PubMed, Embase, Cochrane Library, Web of Science, Google Scholar, and the Chinese Biomedical Literature database (CBM) from 31 December 2019 to 18 June 2020. We applied no language restrictions. We screened abstracts for relevance, extracted data, and assessed the risk of bias in duplicate. We rated the certainty of evidence using the GRADE approach. The primary outcome was severe acute respiratory syndrome coronavirus 2 (SARS-CoV-2) test positivity in neonates born to mothers with confirmed COVID-19 following different delivery modes. Secondary outcomes were neonatal deaths and maternal deaths. This study is registered with PROSPERO, CRD42020194049.

**Results:** Sixty-eight observational studies meeting inclusion criteria were included in the current study, with no randomized controlled trials. In total, information on the mode of delivery, detailed neonatal outcomes, and SARS-CoV-2 status were available for 1,019 pregnant women and 1,035 neonates. Six hundred and eighteen (59.71%) neonates were born through cesarean section and 417(40.29%) through vaginal delivery. Probable congenital SARS-CoV-2 infections were reported in 34/1,035 (3.29%) neonates. Of babies born vaginally, 9/417 (2.16%) were tested positive compared with 25/618 (4.05%) born by cesarean. Of babies born vaginally, 0/417 (0.00%) neonatal deaths were reported compared with 6/618 (0.97%) born by cesarean. Of women who delivered vaginally, 1/416 (0.24%) maternal deaths were reported compared with 11/603 (1.82%) delivered by cesarean. Two women died before delivery. Sensitivity analyses and subgroup analyses showed similar findings.

**Conclusions:** The rate of neonatal COVID-19 infection, neonatal deaths, and maternal deaths are no greater when the mother gave birth through vaginal delivery. Based on the evidence available, there is no sufficient evidence supporting that the cesarean section is better than vaginal delivery in preventing possible vertical transmission from a pregnant mother confirmed with COVID-19 to a neonate. The mode of birth should be individualized and based on disease severity and obstetric indications. Additional good-quality studies with comprehensive serial tests from multiple specimens are urgently needed.

**Study registration**: PROSPERO CRD42020194049.

## Introduction

Since the outbreak of a cluster of patients with pneumonia of unknown cause in Wuhan, Hubei Province, China in December 2019 (1). The disease was later named Coronavirus disease 2019 (COVID-19), caused by the novel severe acute respiratory syndrome coronavirus 2 (SARS-CoV-2) quickly spreading in China and other countries (2).

COVID-19 is the third coronavirus outbreak in the twenty-first century, and the other two are severe acute respiratory syndrome coronavirus (SARS-CoV) outbreaks in 2002 and Middle East respiratory syndrome coronavirus (MERS-CoV) outbreaks in 2012 (3–5), both can cause severe complications during pregnancy (6–9). Pregnant women might be at increased risk of severe infections considering that the COVID-19 seems to have a similar pathogenic potential as SARS-CoV and MERS-CoV. Pregnant women are generally susceptible to COVID-19 considering they are in a particular state of immune suppression and more susceptible to respiratory pathogens (10, 11). Because of decreased lung volumes caused by increases in uterus size during pregnancy, patients might be more prone to have a more rapid clinical deterioration with COVID-19 during pregnancy, which may increase the risk of adverse pregnancy outcomes.

There is a concern about the vertical transmission of SARS-CoV-2 due to the limited data on COVID-19 (12). Until now, vertical transmission from a pregnant mother confirmed with COVID-19 to a neonate, and the delivery mode which can best prevent this from happening is still unknown. Expert consensus has stated that there is no clear evidence that cesarean delivery prevents vertical transmission at the time of delivery (13). Whether vaginal delivery increases the risk of mother-to-child intrapartum transmission and whether uterine contraction could increase the possibility of the virus ascending needs to be further investigated.

Therefore, this review aims to determine which mode of delivery is better for preventing possible vertical transmission from COVID-19 positive pregnant women to the neonate.

## Methods

We wrote the review based on Preferred Reporting Items for Systematic Reviews and Meta-analyses (PRISMA) guidelines (14). The protocol was registered in the International Prospective Register of Systematic Reviews (known as PROSPERO; registration number: CRD42020194049).

### Data Sources, Search Strategy, and Eligibility Criteria

We conducted a comprehensive literature search of PubMed, Embase, Cochrane Library, Web of Science, Google Scholar, and the Chinese Biomedical Literature database (CBM) from 31 December 2019 (when COVID-19 was first reported from Wuhan, China) to 18 June 2020. We also searched the references of selected studies. We placed no limits or filters on the searches. Combinations of the following keywords and MeSH terms were used: 2019-nCov, COVID-19, coronavirus disease 2019, severe acute respiratory syndrome coronavirus 2, SARS-CoV-2, pregnancy, pregnant, gravidity, gestation, maternal, mothers, fetal, fetus, neonate, newborn, vertical transmission, maternal-fetal transmission, intrauterine transmission, delivery. A detailed search strategy can be seen in Appendix 1. Eligibility criteria were randomized controlled studies, observational studies (including cohort, case-control studies, case series, and case reports), studies involving laboratory-confirmed and/or clinically diagnosed COVID-19 during pregnancy, studies involving neonates born to mothers with confirmed COVID-19 infection, studies with available clinical characteristics, including neonatal outcomes, clinical studies, studies reporting original data, studies reporting SARS-CoV-2 infected women who have delivered. Exclusion criteria were as follows: Studies involving mothers with suspected COVID-19 infection, studies with unreported neonatal outcomes, unpublished reports, studies suspected of including duplicate reporting, review, guidelines, opinions, and comments.

Suspected case defined as a person who meets the clinical **AND** epidemiological criteria (15):

### Clinical Criteria

- Acute onset of fever AND cough; OR
- Acute onset of ANY THREE OR MORE of the following signs or symptoms: fever, cough, general weakness/fatigue, headache, myalgia, sore throat, coryza, dyspnoea, anorexia/nausea/vomiting, diarrhea, altered mental status (signs separated with a slash are to be counted as one sign).

### Epidemiological Criteria

- Residing or working in an area with a high risk of transmission of virus: closed residential settings, humanitarian settings such as camp and camp-like settings for displaced persons; anytime within the 14 days prior to symptom onset; OR
- Residing or travel to an area with community transmission anytime within the 14 days prior to symptom onset; OR
- Working in any health care setting, including within health facilities or within the community; any time within the 14 days prior to symptom onset.

A clinically diagnosed case was defined as a suspected case with manifestations with pneumonia image features on computerized tomography (CT) scan (Another potential cause of pneumonia was rule out before diagnosis) (16).

A laboratory-confirmed case was defined as a positive result to reverse transcriptase-polymerase chain reaction (RT-PCR) nasopharyngeal swab and/or antibody testing SARS-CoV-2 (15).

A positive result of antibody testing of SARS-CoV-2 was defined as elevated concentrations of immunoglobulin M (the normal IgM level: <10 AU/mL).

### Study Selection

Two independent reviewers (B.W and H.W) evaluated articles for potential inclusion by screening titles and abstracts. The full texts of those identified as being relevant were assessed to determine eligibility for final inclusion. Between each assessment, we discussed the results to reach a consensus on interpreting the inclusion criteria. We resolved any disagreements regarding study eligibility by consensus, and a third reviewer (D.Z) was consulted, if necessary. If the information required to assess eligibility is unavailable or unclear, the relevant study authors were contacted for clarification. Duplicate publications were identified and removed using EndNote software version X7 (Clarivate Analytics). The identified paper(s) were analyzed using criteria based on the largest sample size, the maximum correspondence with the inclusion criteria, and minimal risk of bias. When a hospital had published their cases more than once, if the periods of recruitment overlapped, we included the paper with the biggest data to minimize the possibility of double counting.

### Data Extraction and Synthesis

We extracted data from the studies selected for inclusion, as follows: general characteristics of included studies (author names, title, publication date, source of funding, and reported conflicts of interests), type of the study, sample size, study subject characteristics (demographic characteristics, gestational age, mode of delivery), outcome measures and analyses (neonatal outcomes, number of positive samples, maternal deaths). Two authors (H.X.Z.and H.L.) extracted the data independently and in duplicate. We resolved discrepancies through discussion to achieve a consensus. Study authors were contacted to obtain missing information or to clarify the information available. However, at the time of submission, we received no responses. The SARS-CoV-2 test positivity in neonates born to mothers with confirmed COVID-19 following different delivery modes was the primary outcome. Secondary outcomes were neonatal deaths and maternal deaths. Our search did not identify any randomized trials. We did a narrative synthesis of the findings when the meta-analysis was not possible or appropriate from the included studies.

### Risk of Bias and Grade Certainty Assessment

The risk of bias was assessed using the Newcastle-Ottawa scale (NOS) for observational cohort and case-control studies, and Joanna Briggs Institute (JBI) critical appraisal tools for case reports and case series studies (17, 18). For cohort and case-control studies, there were three grouping items as follows: selection, comparability, exposure/outcomes. A study can be awarded a maximum of one star for each numbered item within the selection and outcome categories. A maximum of two stars can be given for comparability (17). More stars are equalling lower risk. Case reports and case series studies were categorized according to the percentage of positive answers to each of the questions. Low risk of bias indicated more than 70% of positive answers; moderate risk of bias ranged between 50 and 69%, and high risk of bias represented <49% of positive answers (19). We graded the certainty of evidence using the GRADE approach. We used the GRADEpro guideline development tool (GDT) app to rate evidence and present it in a summary of findings table (20).

### Data Analysis

Characteristics of each study, and results were described and tabulated. We also performed additional sensitivity analyses to assess the robustness of our findings. Sensitivity analyses for the primary outcome and secondary outcomes included: (1) we excluded cases reported neonate with only elevated IgM levels for SARS-CoV-2 but negative for RT-PCR considering the possibility of false-positive results for the serological test (21); (2) we conducted subgroup analyses by pregnant women who were symptomatic or asymptomatic before delivery. Clinically, since an asymptomatic patient identified at screening might not have any delivery complications as opposed to a symptomatic one, where the mode of delivery might be because of maternal indication. Thus, perinatal transmission study might be biased; (3) the infection moment when pregnant women confirmed COVID-19 (i.e., infection on first, second, third trimester). All sensitivity analyses were considered exploratory. No other statistical analyses were anticipated.

## Results

**Figure 1:**
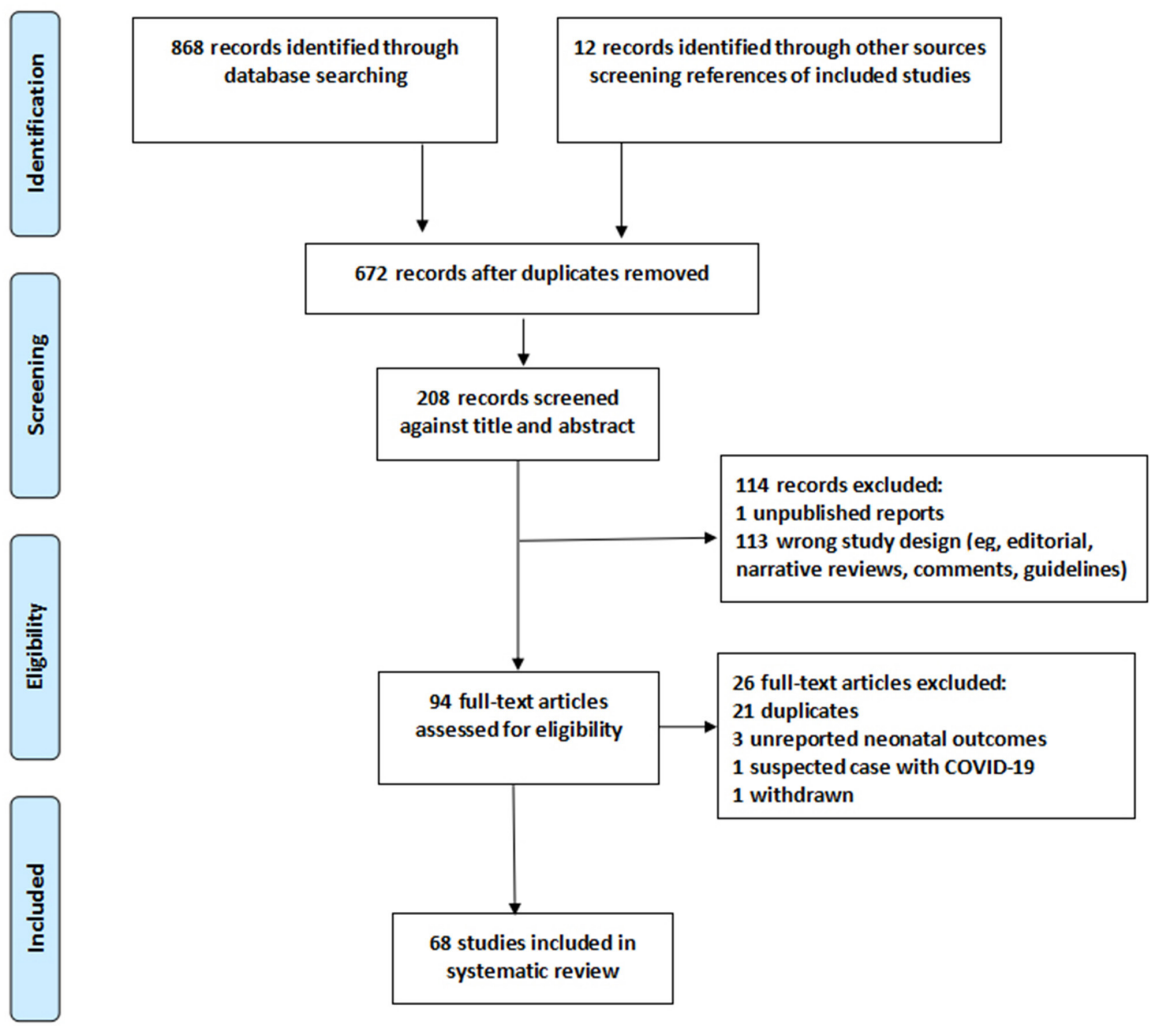
PRISMA flow diagram.

We identified 880 studies, 94 full-text articles assessed for eligibility, including 21 duplicates (cases reported by one or more of the same hospitals and study dates overlap), three unreported neonatal outcomes, one withdrawn at the request of the author, one study reported pregnant women with suspected COVID-19 infection (Figure 1). The detailed information on these excluded studies can be seen in Appendix S7.

In total, sixty-eight studies from 21 countries that meet the eligibility were included in our systematic review. Studies were all observational in nature. We identified no randomized controlled trials in the search strategy. These were published from 6 February 2020 to 12 June 2020. Forty-one were case report studies, 22 were case series studies, four cohort studies, and one case-control study (Table 1).

**Table 1:** Characteristics of the included studies.

**Study**	**Date of publication**	**Country**	**Study design**	**Language of publication**	**Study period**	**No. of pregnant women confirmed with COVID-19 (n)**	**Maternal age, y (range)**	**Gestational age at delivery, week (range)**	**Funding**
Zhu et al. (22)	Feb. 6, 2020	China	Case series	English	1.20~2.05	9	25~35	31~39	No
Li et al. (23)	Feb. 19, 2020	China	Case report	Chinese	1.29~2.02	1	27	38	No
Wang et al. (24)	Feb. 28, 2020	China	Case report	English	2.05~2.18	1	28	30	Yes
Chen et al. (25)	Mar. 2, 2020	China	Case reports	Chinese	1.21~2.25	3	23~34	35~38+6/7	No
Lei et al. (26)	Mar. 2, 2020	China	Case series	Chinese	1.22~2.01	9	29~35	34+2/7~37 +5/7	No
Yao et al. (27)	Mar. 2, 2020	China	Case report	Chinese	2.11~2.25	1	22	38+2/7	No
Zhao et al. (28)	Mar. 6, 2020	China	Case report	Chinese	1.31~2.13	1	Not reported	35+5/7	No
Bai et al. (29)	Mar. 6, 2020	China	Case report	Chinese	2.01~2.15	1	Not reported	37+1/7	No
Kang et al. (30)	Mar. 11, 2020	China	Case report	Chinese	2.06~2.24	1	30	35+4/7	No
Chen et al. (31)	Mar. 16, 2020	China	Case reports	English	Not mention	4	23~34	37+2/7~39	Yes
Zhou et al. (32)	Mar. 16, 2020	China	Case report	Chinese	2.12~2.21	1	30	37+3/7	No
Khan et al. (33)	Mar. 19, 2020	China	Case reports	English	1.28~3.01	3	27~33	34+6/7~39 +1/7	Yes
Li et al. (34)	March 20, 2020	China	Case-control study	English	1.24~2.29	16	26~37	33+6/7~40 +4/7	Yes
Yu et al. (35)	March 24, 2020	China	Case series	English	1.01~2.08	7	29~34	37~41+2/7	Yes
Zeng et al. (36)	Mar. 26, 2020	China	Case series	English	1.30~2.15	33	Not reported	31+2/7~ 40+4/7	No
Zeng et al. (37)	Mar. 26, 2020	China	Case series	English	2.16~3.06	6	Not reported	Not reported	Yes
Dong et al. (38)	Mar. 27, 2020	China	Case report	English	1.28~2.22	1	29	34+2/7	Yes
Chen et al. (39)	Mar. 28, 2020	China	Case series	English	1.20~2.10	5	25~31	38+6/7~40 +4/7	Yes
Baud et al. (40)	Mar. 30, 2020	Switzerland	Case report	English	3.18~3.22	1	28	19	No
Lee et al. (41)	Mar. 31, 2020	Korea	Case report	English	3.06~3.11	1	28	37+6/7	No
Kalafat et al. (42)	Apr. 6, 2020	Turkey	Case report	English	3.20-3.28	1	32	35+3/7	No
Gidlöf et al. (43)	Apr. 6, 2020	Sweden	Case report	English	Not mention	1	34	36+2/7	No
Peng et al. (44)	Apr. 6, 2020	China	Case report	English	2.05~2.19	1	25	35+3/7	No
Breslin et al. (45)	Apr. 9, 2020	USA	Case series	English	3.13~3.27	43	20~39	32~39	No
Xiong et al. (46)	Apr. 10, 2020	China	Case report	English	3.7~3.10	1	25	38+4/7	No
Khassawneh et al. (47)	Apr. 14, 2020	Jordan	Case report	English	3.23~3.26	1	30	36+3/7	No
Schnettler et al. (48)	Apr. 14, 2020	USA	Case report	English	3.24-4.10	1	39	34+1/7	No
Liu et al. (49)	Apr. 14, 2020	China	Case series	English	1.31~2.29	19	26~38	35+2/7~41 +2/7	No
Carosso et al. (50)	Apr. 14, 2020	Italy	Case report	English	Not mention	1	28	37	No
González Romero (51)	Apr. 17, 2020	Spain	Case report	Spanish	Not mention	1	44	29+2/7	No
Koumoutsea et al. (52)	Apr. 17, 2020	Canada	Case report	English	Not mention	2	23~40	35+3/7~35 +5/7	No
Zamaniyan et al. (53)	Apr. 17, 2020	Iran	Case report	English	3.7~3.26	1	22	32	No
Alzamora et al. (54)	Apr. 18, 2020	Peru	Case report	English	3.29~4.03	1	41	33	No
Lyra et al. (55)	Apr. 20, 2020	Portugal	Case report	English	Not mention	1	35	39+6/7	No
Al-kuraishy et al. (56)	Apr. 21, 2020	Iraq	Case report	English	3.13~3.30	1	25	30	No
Lu et al. (57)	Apr. 24, 2020	China	Case report	English	2.11~2.17	1	22	38	Yes
Ferrazzi et al. (58)	Apr. 27, 2020	Italy	Case series	English	3.01~3.20	42	21~44	Not reported	No
Hantoushzadeh et al. (59)	Apr. 28, 2020	Iran	Case series	English	2.15~3.15	9	Not reported	28~38+3/7	Yes
Penfield et al. (60)	May 3, 2020	USA	Case series	English	3.01~4.20	32	22~40	26+5/7~41 +3/7	No
Wu et al. (61)	May 5, 2020	China	Case series	English	1.31~3.09	13	26~40	16~38+4/7	Yes
Piersigilli et al. (62)	May 7, 2020	Belgium	Case report	English	3.01~3.15	1	Not reported	26+4/7	No
Blauvelt et al. (63)	May 8, 2020	USA	Case report	English	Not mention	1	34	28+6/7	Yes
Pierce-Williams et al. (64)	May 8, 2020	USA	Cohort study	English	4.20~5.05	64	Not reported	Not reported	No
Valente et al. (65)	May 10, 2020	Portugal	Case report	English	3.17~3.19	1	31	38	No
Liu et al. (66)	May 11, 2020	China	Case series	English	1.20~3.03	51	Not reported	35+1/7~41 +2/7	Yes
Perrone et al. (67)	May 11, 2020	Italy	Case reports	English	3.01~4.30	4	26~36	38+2/7~40 +4/7	No
Baergen et al. (68)	May 12, 2020	USA	Case series	English	Not mention	20	16~41	32+2/7~40 +4/7	No
Taghizadieh et al. (69)	May 13, 2020	Iran	Case report	English	Not mention	1	33	34	No
Patanè et al. (70)	May 14, 2020	Italy	Case series	English	3.05~4.21	22	Not reported	Not reported	No
Kirtsman et al. (71)	May 14, 2020	Canada	Case report	English	Not mention	1	40	35+5/7	No
Dória et al. (72)	May 15, 2020	Portugal	Case series	English	3.25~4.15	10	27~40	37~41	No
Mehta et al. (73)	May 16, 2020	USA	Case report	English	Not mention	1	39	27	No
Chen et al. (74)	May 16, 2020	China	Case series	English	Not mention	17	Not reported	Not reported	No
Sharma et al. (75)	May 17, 2020	India	Case report	English	Not mention	1	Not reported	38+6/7	No
Xia et al. (76)	May 17, 2020	China	Case report	English	1.23~2.20	1	27	37+2/7	Yes
Panichaya et al. (77)	May 18, 2020	Thailand	Case report	English	Not mention	1	43	18	No
Lokken et al. (78)	May 18, 2020	USA	Case series	English	1.21~4.17	46	26~34	37+3/7~39 +6/7	Yes
London et al. (79)	May 19, 2020	USA	Cohort study	English	3.15~4.15	68	24~34	Not reported	No
Li et al. (80)	May 19, 2020	China	Case report	English	2.06~2.19	1	30	35	No
Qadri et al. (81)	May 20, 2020	USA	Case series	English	Not mention	16	20~40	22~40+3/7	No
Tang et al. (82)	May 23, 2020	Netherlands	Case report	English	4.01~4.30	1	Not reported	41	No
Lowe et al. (83)	May 28, 2020	Australia	Case report	English	Not mention	1	31	40+3/7	No
Kayem et al. (84)	Jun. 4, 2020	France	Case series	English	3.01~4.14	617	Not reported	Not reported	No
Martínez-Perez et al. (85)	Jun. 8, 2020	Spain	Cohort study	English	3.12~4.06	82	19~48	25~41+4/7	Yes
Wang et al. (86)	Jun. 8, 2020	China	Case series	English	12.08~4.01	30	26~33	30~40+6/7	No
Knight et al. (87)	Jun. 8, 2020	UK	Cohort study	English	3.01~4.14	427	Not reported	Not reported	Yes
Pereira et al. (88)	Jun. 10, 2020	Spain	Case series	English	3.14~4.14	60	22~43	27~41	No
Bani Hani et al. (89)	Jun. 12, 2020	Jordan	Case report	English	3.28~4.12	1	29	37+4/7	No

Among the five comparative studies (cohort and case-control), one study compared maternal and neonatal outcomes of pregnant women with and without COVID-19 infections. Three studies compared the clinical course of pregnant women with mild, severe, or critical COVID-19 pneumonia. Only one cohort study estimates associations between delivery mode (vaginal vs. cesarean delivery) and maternal and neonatal outcomes among SARS-CoV-2–infected women giving birth. It was impossible to perform a meta-analysis in this systematic review. Thus, we did a narrative synthesis of the findings from the included studies.

The maternal age of the reported cases ranged from 16 to 48 years, gestational age at diagnosis ranged from 16 to 41 weeks. A total of 1,019 women and 1,035 neonates had detailed information on the delivery mode and infant infection status, including 14 sets of twins and one set of triplets. Among fifteen multiple pregnancies, one woman had a vaginal birth for twins, and the others had a cesarean section.

Of the 1,035 neonates, 618 (59.71%) were born through cesarean section and 417 (40.29%) through vaginal delivery (Table 2). SARS-CoV-2 infections were reported in 34/1,035 (3.29%) neonates, included thirty-one RT-PCR positive neonates. The other three neonates had elevated levels of IgM for SARS-CoV-2 but negative for RT-PCR. Of the 416 women who delivered vaginally, 9/417 (2.16%) neonates tested positive for COVID-19. Of the 603 women who had a cesarean section, 25/618 (4.05%) neonates were found to be positive for COVID-19. A total of six neonatal deaths (including one set of twins) and nine stillbirths (including one set of twins) have been reported. Of babies born vaginally, 0/417 (0.00%) neonatal deaths were reported compared with 6/618 (0.97%) born by cesarean. A total of fourteen maternal deaths have been reported. Of women who delivered vaginally, 1/416 (0.24%) maternal deaths were reported compared with 11/603 (1.82%) delivered by cesarean. Two women died in the second trimester before delivery.

**Table 2:** Maternal and neonatal outcomes by mode of delivery.

	**Vaginal delivery**	**Cesarean delivery**
	**No. (%) (*n* = 416)^**a**^**	**No. (%) (*n* = 603)^**b**^**
**Comorbidities**^**c**^	32 (29.36%)	60 (27.91%)
**INDICATIONS FOR CESAREAN DELIVERY**^**d**^		
Due to obstetrical indications	NA	407 (68.52%)
Due to concern about Covid-19	NA	187 (31.48%)
**MATERNAL OUTCOMES**		
Maternal deaths^e^ (secondary outcome)	1 (0.24%)	11 (1.82%)
**NEONATAL OUTCOMES**		
SARS-CoV-2 test positivity^f^ (primary outcome)	9 (2.16%)	25 (4.05%)
Neonatal deaths^g^ (secondary outcome)	0 (0.00%)	6 (0.97%)

*SARS-CoV-2 = severe acute respiratory syndrome coronavirus 2; NA, Not applicable*.

a *416 pregnant women gave birth vaginally, including one set of twins*.

b *603 women gave birth by cesarean section, including one set of triplets and thirteen sets of twins*.

c *Detailed information on comorbidities was available for 324 pregnant women. Of the 109 women who delivered vaginally, 32 had one or more comorbidities. Of the 215 women who had a cesarean delivery, 60 had one or more comorbidities*.

d *Detailed information on the indication for cesarean section was available for 594 pregnant women*.

e *Excluding two women who died in the second trimester before delivery*.

f *Including 31 reverse transcriptase-polymerase chain reaction positive neonates and three elevated Immunoglobulin M levels for SARS-CoV-2 neonates*.

g *Excluding nine stillbirths*.

The risk of bias was mostly low-to-moderate after considering the observational designs. The results for each quality assessment by the study are presented in Appendix S2–S4.

Sensitivity analyses and subgroup analyses showed similar findings (Appendix S6). After excluded 3 (All three neonates were born through cesarean section) elevated levels of IgM for SARS-CoV-2 but negative for RT-PCR, 9/417 (2.16%) neonates born by vaginally tested positive compared with 22/615 (3.58%) neonates born by cesarean. Of the 394 women who were asymptomatic before delivery, 0/220 (0%) maternal deaths were reported with vaginal delivery compared with 2/174 (1.15%) with cesarean delivery. Of the 625 women who were symptomatic before delivery, 1/196 (0.51%) maternal deaths with vaginal delivery were reported compared with 9/429 (2.10%) with cesarean delivery. Nearly all pregnant women delivered in the third trimester except three who delivered vaginally in the second trimester. Of women who delivered vaginally in the third trimester, 1/413 (0.24%) maternal deaths were reported compared with 11/603 (1.82%) delivered by cesarean in the third trimester.

## Discussion

Our results have shown that SARS-CoV-2 infections were rare in neonates. The rate of neonatal COVID-19 infection, neonatal deaths, and maternal deaths is no greater when the mother gave birth through vaginal delivery. Second, the vertical transmission of SARS-CoV-2 infection is possible in the third trimester but relatively low. Third, there has been duplicate reporting of pregnant women confirmed with COVID-19 from China and other countries.

Vertical transmission refers to how pathogens are transmitted from mother to offspring before and after birth. It includes transmission via placental blood during pregnancy, via the birth canal during delivery, and via breastmilk during postpartum breastfeeding (22). Placenta, cord blood, amniotic fluid, and vaginal secretion are intrauterine tissue samples that are essential for assessing vertical transmission (90). It is necessary to collect more kinds of specimens of SARS-CoV-2 infected pregnant women and their newborns to better evaluating the possibility of vertical transmission of SARS-CoV-2. It is noteworthy that these samples should be collected immediately after birth to avoid contamination condition (91). But very few of the included studies have met these criteria. Thus, additional good-quality studies with comprehensive serial tests from multiple specimens are urgently needed.

A total of 34 neonates were born in the third trimester with possible congenital SARS-CoV-2 infections were reported, suggesting that vertical transmission of COVID-19 is possible in the third trimester. Only three pregnant women gave birth vaginally in the second trimester. All neonates' samples tested negative by RT-PCR, suggesting that no intrauterine fetal infection occurred during the second trimester of pregnancy. A recent study suggested that the SARS-CoV-2-infected mother-to-fetus transmission ratio will be significantly lower than that of the Zika virus. Because the expression of angiotensin-converting enzyme 2 (ACE2), which is the receptor that SARS-CoV-2 enters the cell, is deficient in all kinds of early maternal-fetal interface cells (92). And this may explain why SARS-CoV-2 can be found in human saliva rather than in vaginal secretions (93), which could also partially explain why the risk of intrauterine mother-to-child transmission for SARS-CoV-2 is low.

Thirty-one neonates tested positive for SARS-CoV-2 by RT-PCR. The remaining three neonates had elevated IgM levels for SARS-CoV-2 but negative by RT-PCR. These three cases deserve additional details. Two studies reported three neonates with elevated IgM antibody values to SARS-CoV-2 born to mothers with COVID-19 from separate research teams in China (37, 94). All mothers wore masks during the cesarean delivery in negative pressure isolation rooms, and all medical staff wore protective suits and double masks. After birth, all infants were isolated from their mothers immediately. Neonatal blood was collected to test IgG and IgM antibodies to SARS-CoV-2 at 0 and 2 h after birth, respectively. However, all of the three neonatal respiratory samples tested negative for SARS-CoV-2 RNA, and there was no information provided by testing cord blood or placenta.

It is worth noting that IgM antibodies are not usually transferred from mother to fetus via the placenta because of the larger macromolecular structure (95). IgM is generally the first responded antibody that eliminating pathogens before sufficient IgG is produced (96). IgM positive results tend to indicate recent exposure to SARS-CoV-2. In contrast, the detection of COVID-19 IgG antibodies means virus exposure some time ago. IgM antibodies usually take days to appear after infection. IgM antibodies can be detected after a median of 5 days following the onset of symptoms (97). Most guidelines using nucleic acid tests as the gold standard for the diagnosis of COVID-19 due to the time lag between the onset of symptoms and IgM's appearance in serum and a lower sensitivity and specificity of serological tests (98, 99). Caution in interpreting these findings has been suggested, including the possibility of false-positive results (100). Thus, sensitivity analyses were performed by excluded reported elevated levels of IgM for SARS-CoV-2 but negative for RT-PCR. Sensitivity analyses showed the rate of neonatal COVID-19 infection still lower when the baby is born vaginally. Additional two sensitivity analyses showed similar findings regarding the moment of the infection and if pregnant women were symptomatic or asymptomatic before delivery.

Most of the guidelines for managing pregnant women with COVID-19 are based on previous SARS and MERS experience (13, 101, 102). Suggestions on the selection of delivery methods in pregnancies with COVID-19 are contradictory (13). There were no confirmed cases of vertical transmission for SARS-CoV and MERS (103). Despite causing approximately one billion annual infections globally, the influenza virus has only a few cases of confirmed or suspected intrauterine fetal infections reported (104). Evidence for intrauterine influenza transmission exists from antigen and antibody testing in the infant brain, amniotic fluid, fetal heart, and cord blood (105). Nevertheless, which delivery mode is better for preventing vertical transmission from a pregnant woman with influenza to a neonate remains unknown.

While we have presented the data from a robust search of the literature for 1,019 women and 1,035 neonates, the given number did not control some confounding factors. For example, the mothers' COVID-19 infection severity was not presented due to the missing information of the included studies. What's more, the baseline conditions of pregnant women undergoing cesarean section and vaginal delivery are different, so we remind the reader to interpret the data in light of these biases would weaken the conclusions of current studies. The overall rate of cesarean section in the included studies was 59.18% (603/1,019), much higher than cesarean birth rates in the United States (31.9%) and China (36.7%) (106, 107). About 31.48% (187/594) of the cesarean deliveries were performed among women with COVID-19 due to concern about Covid-19 without obstetrical indications. According to a WHO report, (108) the rates of complications during pregnancy were similar between women who delivered vaginally (18.36%) or by cesarean section (19.57%). The neonatal death rates were similar between babies born vaginally (0.59%) or by cesarean section (0.79%). However, for maternal mortality, cesarean sections were associated with a significantly increased risk of maternal mortality than vaginal delivery (adjusted odds ratio 2.1, 95% CI 1.7–2.6). Furthermore, cesarean delivery is associated with increased morbidity in the immediate postpartum period because of the increased risks of thromboembolic disease, blood loss, and infections (109). Currently, there is no sufficient evidence supporting that cesarean section improves outcomes among patients with COVID-19 and prevent possible vertical transmission from a pregnant mother confirmed with COVID-19 to a neonate. Our findings suggest that COVID-19 infection should not be an indication for a cesarean birth. We advise that cesarean delivery be performed in women with COVID-19 only after a careful evaluation of the disease severity and obstetrical indications. We believe our findings are reassuring and relevant to pregnant women confirmed with COVID-19 and obstetricians. Especially pregnant women with COVID-19 who want to give birth by vaginal delivery.

We identified 21 duplicate studies, some articles have been published in different languages, and some authors have reported features of pregnant women with COVID-19 from different perspectives. The detailed information on these duplicates studies can be seen in Appendix S7. There have several concerns about duplicate reporting of cases of COVID-19 been described (110, 111). Reporting the duplicates in different articles creates an inaccurate scientific record, precludes valid meta-analyses considering double-counting, and may affect understanding the disease and its epidemiology (110). To minimize the possibility of double counting, we reviewed the hospital and periods of recruitment. If they overlapped, only the study with the biggest data was included.

## Limitations of Study

Our article has some limitations. Firstly, we didn't search for the LILACS or SciELO database, which means the data on pregnant women from Latin America, the Caribbean region, and Brazil are scarce. Brazil has the third-largest number of COVID-19 cases after the United States and India. Secondly, we didn't perform analysis according to the severity of the COVID-19 infection of the mothers due to the missing information of the included studies. Pregnant women with more severe COVID-19 infection appear to prefer delivery by cesarean delivery rather than vaginal birth (112, 113). What's more, all patients in the study who give birth were recruited in their second and third trimester, so we were unable to ascertain the possibility of intrauterine vertical transmission during the first trimester. For example, rubella infection in the first trimester can affect more than 50% of fetuses via intrauterine infection. In contrast, by the end of the second trimester, the incidence rate is reduced by half (114).

## Conclusions

The rate of neonatal COVID-19 infection, neonatal deaths, and maternal deaths is no greater when the mother gave birth through vaginal delivery. Based on the evidence available, there is no sufficient evidence supporting that the cesarean section is better than vaginal delivery in preventing possible vertical transmission from a pregnant mother confirmed with COVID-19 to a neonate. The mode of birth should be individualized and based on disease severity and obstetric indications. Additional good-quality studies with comprehensive serial tests from multiple specimens are urgently needed.

## Data Availability Statement

The original contributions presented in the study are included in the article/Supplementary Material, further inquiries can be directed to the corresponding authors.

## Author Contributions

JC had the idea for the article. DZ and HL contributed to the design of the search strategy. HW and BW did data selection. YY and RZ had roles in the assessment of the risk of bias in the included studies. All authors reviewed and approved the final version.

## Conflict of Interest

The authors declare that the research was conducted in the absence of any commercial or financial relationships that could be construed as a potential conflict of interest.
