# Supplementary material for: Cesarean Section or Vaginal Delivery to Prevent Possible Vertical Transmission From a Pregnant Mother Confirmed With COVID-19 to a Neonate: A Systematic Review
Source: Front Med (Lausanne). 2021 Feb 17;8:634949. doi: 10.3389/fmed.2021.634949 (PMC7926203; doi:10.3389/fmed.2021.634949)
Supplement: Supplementary file 1 [file Data_Sheet_1.DOCX]

**Contents:**

**1) Search strategies.**

**2) Newcastle-Ottawa scale to rate risk of bias for cohort and case-control**

**3) Joanna Briggs Institute critical appraisal tools to rate risk of bias for case reports**

**4) Joanna Briggs Institute critical appraisal tools to rate risk of bias for case series**

**5) Summary of findings table**

**6) Results of the sensitivity analyses for the primary outcome and secondary outcomes**

**7) Excluded studies**

**8) References for the Supplementary material**

**Appendix S1: Search strategies.**

**PubMed**

**Search** **Query**

#1 Search: (2019-nCoV) OR (nCoV-2019) OR (2019-novel coronavirus) OR (2019 novel coronavirus) OR (2019 coronavirus) OR (coronavirus disease 2019) OR (Novel coronavirus pneumonia) OR (NCP) OR (Novel coronavirus) OR (Severe Acute Respiratory Syndrome Coronavirus 2) OR (SARS-CoV-2) OR (COVID-19) OR (coronavirus disease) OR (coronavirus disease-19)

#2 Search: (Pregnancy[mh]) OR (Pregnancies) OR (Gestation)

#3 Search: (Fetus[mh]) OR (Fetuses) OR (Fetal Structures) OR (Fetal Structure) OR (Structure, Fetal) OR (Structures, Fetal) OR (Mummified Fetus) OR (Fetus, Mummified) OR (Retained Fetus) OR (Fetus, Retained) OR (Fetal Tissues) OR (Fetal Tissue) OR (Tissue, Fetal) OR (Tissues, Fetal)

#4 Search: (Infant, Newborn[mh]) OR (Infants, Newborn) OR (Newborn Infant) OR (Newborn Infants) OR (Newborn) OR (Newborns) OR (Neonate) OR (Neonates)

#5 Search: #1 and #2

#6 Search: #3 or #4

#7 Search: #5 and #6

**Embase**

**No.** **Query**

#1 'coronavirus disease 2019'/exp

#2 'pregnancy'/exp

#3 'newborn'/exp

#4 'fetus'/exp

#5 #1 and #2

#6 #3 or #4

#7 #5 and #6

**Cochrane Library**

**ID Search Hits**

#1 MeSH descriptor: [Pregnancy] explode all trees 21850

#2 MeSH descriptor: [Coronavirus] explode all trees 72

#3 COVID 1577

#4 nCov 81

#5 severe acute respiratory syndrome coronavirus 2 381

#6 SARS-CoV-2 96

#7 NCP 110

#8 novel coronavirus 200

#9 MeSH descriptor: [Fetus] explode all trees 1754

#10 MeSH descriptor: [Infant] explode all trees 31836

#11 #2 OR #3 OR #4 OR #5 OR #6 OR #7 OR #8 1770

#12 #1 AND #11 0

#13 #9 OR #10 32633

#14 #12 AND # 13 0

**Web of Science** (Index=SCI-EXPANDED, SSCI, A&HCI, ESCI)

# 19 #18 AND #17 187

# 18 #8 AND #4 542

# 17 #16 OR #12 285,217

# 16 #15 OR #14 OR #13 186,138

# 15 KP=((Infant, Newborn[mh]) OR (Infants, Newborn) OR (Newborn Infant) OR (Newborn Infants) OR (Newborn) OR (Newborns) OR (Neonate) OR (Neonates) ) 25,687

# 14 AB=((Infant, Newborn[mh]) OR (Infants, Newborn) OR (Newborn Infant) OR (Newborn Infants) OR (Newborn) OR (Newborns) OR (Neonate) OR (Neonates) ) 136,969

# 13 TI=((Infant, Newborn[mh]) OR (Infants, Newborn) OR (Newborn Infant) OR (Newborn Infants) OR (Newborn) OR (Newborns) OR (Neonate) OR (Neonates) ) 67,879

# 12 #11 OR #10 OR #9 113,234

# 11 KP=((Fetus[mh]) OR (Fetuses) OR (Fetal Structures) OR (Fetal Structure) OR (Structure, Fetal) OR (Structures, Fetal) OR (Mummified Fetus) OR (Fetus, Mummified) OR (Retained Fetus) OR (Fetus, Retained) OR (Fetal Tissues) OR (Fetal Tissue) OR (Tissue, Fetal) OR (Tissues, Fetal) ) 15,952

# 10 AB=((Fetus[mh]) OR (Fetuses) OR (Fetal Structures) OR (Fetal Structure) OR (Structure, Fetal) OR (Structures, Fetal) OR (Mummified Fetus) OR (Fetus, Mummified)

OR (Retained Fetus) OR (Fetus, Retained) OR (Fetal Tissues) OR (Fetal Tissue) OR (Tissue, Fetal) OR (Tissues, Fetal) ) 95,835

# 9 TI=((Fetus[mh]) OR (Fetuses) OR (Fetal Structures) OR (Fetal Structure) OR (Structure, Fetal) OR (Structures, Fetal) OR (Mummified Fetus) OR (Fetus, Mummified) OR (Retained Fetus) OR (Fetus, Retained) OR (Fetal Tissues) OR (Fetal Tissue) OR (Tissue, Fetal) OR (Tissues, Fetal) ) 19,060

# 8 #7 OR #6 OR #5 438,731

# 7 KP=((Pregnancy[mh]) OR (Pregnancies) OR (Gestation) ) 120,796

# 6 AB=((Pregnancy[mh]) OR (Pregnancies) OR (Gestation) ) 309,967

# 5 TI=((Pregnancy[mh]) OR (Pregnancies) OR (Gestation) ) 161,680

# 4 #3 OR #2 OR #1 53,505

# 3 KP=((2019-nCoV) OR (nCoV-2019) OR (2019-novel coronavirus) OR (2019 novel coronavirus) OR (2019 coronavirus) OR (coronavirus disease 2019) OR (Novel coronavirus pneumonia) OR (NCP) OR (Novel coronavirus) OR (Severe Acute Respiratory Syndrome Coronavirus 2) OR (SARS-CoV-2) OR (COVID-19) OR (coronavirus disease) OR (coronavirus disease-19) ) 2,198

# 2 TI=((2019-nCoV) OR (nCoV-2019) OR (2019-novel coronavirus) OR (2019 novel coronavirus) OR (2019 coronavirus) OR (coronavirus disease 2019) OR (Novel coronavirus pneumonia) OR (NCP) OR (Novel coronavirus) OR (Severe Acute Respiratory Syndrome Coronavirus 2) OR (SARS-CoV-2) OR (COVID-19) OR (coronavirus disease) OR (coronavirus disease-19) ) 42,311

# 1 AB=((2019-nCoV) OR (nCoV-2019) OR (2019-novel coronavirus) OR (2019 novel coronavirus) OR (2019 coronavirus) OR (coronavirus disease 2019) OR (Novel coronavirus pneumonia) OR (NCP) OR (Novel coronavirus) OR (Severe Acute Respiratory Syndrome Coronavirus 2) OR (SARS-CoV-2) OR (COVID-19) OR (coronavirus disease) OR (coronavirus disease-19) ) 33,159

**Google Scholar - keywords searching**

2019-nCov, COVID-19, coronavirus disease 2019, severe acute respiratory syndrome coronavirus 2, SARS-CoV-2, pregnancy, gestation, maternal, mothers, vertical transmission, maternal-fetal transmission, intrauterine transmission, delivery.

**Chinese Biomedical Literature database (CBM) - field searching in Chinese**

("2019冠状病毒"[常用字段:智能] OR "新型冠状病毒"[常用字段:智能] OR "新冠肺炎"[常用字段:智能] OR "新冠"[常用字段:智能] OR "2019-nCoV"[常用字段:智能] OR "SARS-CoV-2"[常用字段:智能] OR "Novel coronavirus"[常用字段:智能] OR "nCoV"[常用字段:智能] OR "Coronavirus disease"[常用字段:智能] OR "new coronavirus"[常用字段:智能] OR "COVID-19"[常用字段:智能] OR "coronavirus"[常用字段:智能] AND ( "pregnancy"[常用字段] OR "孕妇"[常用字段] OR "产妇"[常用字段])) AND 2019-2020[日期]

**Appendix S2: Newcastle-Ottawa scale to rate risk of bias for cohort and case-control.**

| **Study** | **Selection** | **Comparability** | **Exposure/Outcome** | **Overall Rating**  **(more stars = lower risk of bias)** |
| --- | --- | --- | --- | --- |
| Li^13^ | ★★ | ★ | ★★ | ★★★★★ |
| Pierce-Williams^43^ | ★★ | -^*^ | ★★ | ★★★★ |
| London^58^ | ★★ | ★ | ★ | ★★★★ |
| Martínez-Perez^64^ | ★★ | ★ | ★ | ★★★★ |
| Knight^66^ | ★★★ | ★ | ★ | ★★★★★ |

^*^A single dash (-) indicates no stars. More stars equalling lower risk.

**Appendix S3: Joanna Briggs Institute critical appraisal tools to rate risk of bias for case reports.**

| **Study** | **Q1** | **Q2** | **Q3** | **Q4** | **Q5** | **Q6** | **Q7** | **Q8** | **Overall appraisal** |
| --- | --- | --- | --- | --- | --- | --- | --- | --- | --- |
| Li^2^ | Yes | No | No | Yes | Yes | Yes | No | Yes | 5/8 |
| Wang^3^ | Yes | Yes | No | Yes | Yes | Yes | No | Yes | 6/8 |
| Chen^4^ | Yes | No | No | Yes | Yes | Yes | No | Yes | 5/8 |
| Yao^6^ | Yes | Yes | Yes | Yes | Yes | No | No | Yes | 6/8 |
| Zhao^7^ | Yes | Yes | Yes | Yes | No | No | No | Yes | 5/8 |
| Bai^8^ | Yes | No | Yes | Yes | Yes | No | No | Yes | 5/8 |
| Kang^9^ | Yes | Yes | No | Yes | No | No | No | Yes | 4/8 |
| Chen^10^ | Yes | No | No | Yes | Yes | Yes | No | Yes | 5/8 |
| Zhou^11^ | Yes | Yes | Yes | Yes | No | No | NA | Yes | 5/8 |
| Khan^12^ | Yes | Yes | No | Yes | Yes | No | No | Yes | 5/8 |
| Dong^17^ | Yes | Yes | Yes | Yes | Yes | No | No | unclear | 5/8 |
| Baud^19^ | Yes | Yes | Yes | Yes | No | No | No | Yes | 5/8 |
| Lee^20^ | Yes | Yes | Yes | Yes | Yes | No | No | Yes | 6/8 |
| E. Kalafat^21^ | Yes | No | Yes | Yes | Yes | No | No | Yes | 5/8 |
| Gidlöf^22^ | Yes | Yes | No | Yes | No | Yes | No | Yes | 5/8 |
| Peng^23^ | Yes | Yes | No | Yes | No | Yes | No | Yes | 5/8 |
| Xiong^25^ | Yes | Yes | Yes | Yes | Yes | Yes | No | Yes | 7/8 |
| Khassawneh^26^ | No | Yes | Yes | Yes | Yes | Yes | No | Yes | 6/8 |
| Schnettler^27^ | Yes | Yes | Yes | Yes | Yes | No | No | Yes | 6/8 |
| Carosso^29^ | Yes | No | Yes | Yes | Yes | No | No | Yes | 5/8 |
| González Romero^30^ | Yes | Yes | No | Yes | No | No | No | Yes | 4/8 |
| Koumoutsea^31^ | Yes | Yes | Yes | Yes | Yes | No | No | Yes | 6/8 |
| Zamaniyan^32^ | Yes | Yes | Yes | Yes | Yes | Yes | Yes | Yes | 8/8 |
| Alzamora^33^ | Yes | Yes | Yes | Yes | Yes | No | No | Yes | 6/8 |
| Lyra^34^ | Yes | No | Yes | Yes | Yes | No | No | Yes | 5/8 |
| Al-kuraishy^35^ | Yes | Yes | Yes | Yes | Yes | Yes | No | Yes | 7/8 |
| Lu^36^ | Yes | Yes | Yes | Yes | No | No | No | Yes | 5/8 |
| Piersigilli^41^ | No | Yes | Yes | Yes | Yes | No | No | Yes | 5/8 |
| Blauvelt^42^ | Yes | No | Yes | Yes | Yes | Yes | No | Yes | 6/8 |
| Valente^44^ | Yes | No | Yes | Yes | Yes | Yes | No | Yes | 6/8 |
| Perrone^46^ | Yes | Yes | Yes | Yes | Yes | No | No | Yes | 6/8 |
| Taghizadieh^48^ | No | Yes | Yes | Yes | Yes | No | No | Yes | 5/8 |
| Kirtsman^50^ | Yes | Yes | Yes | Yes | Yes | No | No | Yes | 6/8 |
| Mehta^52^ | Yes | No | No | Yes | Yes | Yes | No | Yes | 5/8 |
| Sharma^54^ | Yes | No | No | Yes | Yes | No | No | Yes | 4/8 |
| Xia^55^ | Yes | No | Yes | Yes | Yes | No | No | Yes | 5/8 |
| Panichaya^56^ | Yes | No | Yes | Yes | Yes | No | No | No | 4/8 |
| Li^59^ | Yes | Yes | Yes | Yes | Yes | Yes | No | Yes | 7/8 |
| Tang^61^ | No | Yes | Yes | Yes | Yes | Yes | No | Yes | 6/8 |
| Lowe^62^ | Yes | No | Yes | Yes | Yes | No | No | Yes | 5/8 |
| Bani Hani^68^ | Yes | Yes | Yes | Yes | Yes | No | No | Yes | 6/8 |

NA=Not applicable;

Q1: Were patient’s demographic characteristics clearly described? Q2: Was the patient’s history clearly described and presented as a timeline? Q3: Was the current clinical condition of the patient on presentation clearly described? Q4: Were diagnostic tests or assessment methods and the results clearly described? Q5: Was the intervention(s) or treatment procedure(s) clearly described? Q6: Was the post-intervention clinical condition clearly described? Q7: Were adverse events (harms) or unanticipated events identified and described? Q8: Does the case report provide takeaway lessons?

**Appendix S4: Joanna Briggs Institute critical appraisal tools to rate risk of bias for case series.**

| **Study** | **Q1** | **Q2** | **Q3** | **Q4** | **Q5** | **Q6** | **Q7** | **Q8** | **Q9** | **Q10** | **Overall appraisal** |
| --- | --- | --- | --- | --- | --- | --- | --- | --- | --- | --- | --- |
| Zhu^1^ | Yes | Yes | Yes | Yes | No | Yes | Yes | No | Yes | Yes | 8/10 |
| Lei^5^ | Yes | Yes | Yes | Yes | No | Yes | Yes | Yes | No | Yes | 8/10 |
| Yu^14^ | Yes | Yes | Yes | No | No | Yes | Yes | No | Yes | Yes | 7/10 |
| Zeng^15^ | Yes | Yes | Yes | Yes | Unclear | No | Yes | No | NA | Yes | 6/10 |
| Zeng^16^ | Yes | Yes | No | No | Unclear | No | No | No | NA | Yes | 3/10 |
| Chen^18^ | Yes | Yes | Yes | Unclear | Unclear | No | Yes | No | Yes | Yes | 6/10 |
| Breslin^24^ | Yes | Yes | Yes | yes | Unclear | No | Yes | No | No | Yes | 6/10 |
| Liu^28^ | Yes | Yes | Yes | Yes | No | No | Yes | No | No | Yes | 6/10 |
| Ferrazzi^37^ | Yes | Yes | Yes | Unclear | Unclear | No | Yes | No | Yes | Yes | 6/10 |
| Hantoushzadeh^38^ | Yes | Yes | No | Unclear | Unclear | No | Yes | Yes | Yes | Yes | 6/10 |
| Penfield^39^ | Yes | Yes | Yes | Unclear | No | No | No | No | Yes | Yes | 5/10 |
| Wu^40^ | Yes | Yes | Yes | Yes | No | Yes | Yes | No | Yes | Yes | 8/10 |
| Liu^45^ | Yes | Yes | Yes | Unclear | No | Yes | Yes | No | Yes | Yes | 7/10 |
| Baergen^47^ | Yes | Yes | Yes | Unclear | Unclear | No | Yes | No | Yes | Yes | 6/10 |
| Patanè^49^ | Yes | Yes | Yes | Unclear | Unclear | Yes | Yes | No | Yes | Yes | 7/10 |
| Dória^51^ | Yes | Yes | Yes | Unclear | Unclear | Yes | No | No | No | Yes | 5/10 |
| Chen^53^ | Yes | Yes | Yes | Unclear | No | Yes | No | No | Yes | Yes | 6/10 |
| Lokken^57^ | Yes | Yes | Yes | Unclear | No | Yes | Yes | No | Yes | Yes | 7/10 |
| Qadri^60^ | Yes | Yes | Yes | Yes | No | Yes | Yes | No | Yes | Yes | 8/10 |
| Kayem^63^ | Yes | Yes | Yes | Yes | Yes | Yes | Yes | No | Yes | Yes | 9/10 |
| Wang^65^ | Yes | Yes | Yes | Yes | Unclear | Yes | Yes | No | Yes | Yes | 8/10 |
| Pereira^67^ | Yes | Yes | Yes | Yes | Unclear | Yes | Yes | Yes | No | Yes | 8/10 |

NA=Not applicable;

Q1: Were there clear criteria for inclusion in the case series? Q2: Was the condition measured in a standard, reliable way for all participants included in the case series? Q3: Were valid methods used for identification of the condition for all participants included in the case series? Q4: Did the case series have consecutive inclusion of participants? Q5: Did the case series have complete inclusion of participants? Q6: Was there clear reporting of the demographics of the participants in the study? Q7: Was there clear reporting of clinical information of the participants? Q8: Were the outcomes or follow up results of cases clearly reported? Q9: Was there clear reporting of the presenting site(s)/clinic(s) demographic information? Q10: Was statistical analysis appropriate?

**Appendix S5: Summary of findings table.**

| **Which mode of delivery is better for preventing possible vertical transmission from a pregnant mother confirmed with COVID-19 to a neonate? Cesarean or vaginal delivery?** | | | | | | |
| --- | --- | --- | --- | --- | --- | --- |
| **Patient or population**: pregnant women confirmed with COVID-19 to prevent a possible vertical transmission; **Setting**: Hospital ; **Intervention**: Cesarean section ; **Comparison**: vaginal delivery | | | | | | |
| Outcomes | **Anticipated absolute effects^*^** (95% CI) | | Relative effect (95% CI) | № of participants  (studies) | Certainty of the evidence (GRADE) | Comments |
|  | **Risk with vaginal delivery** | **Risk with Cesarean section** |  |  |  |  |
| SARS-CoV-2 test positivity in neonates | Detailed neonatal outcomes and SARS-CoV-2 status were available for 1035 neonates born to mothers with confirmed COVID-19 infection. 618 neonates were born through cesarean section and 417 through vaginal delivery. | |  | (68 observational studies) | ⨁⨁◯◯ LOW | In all, SARS-CoV-2 infections were reported in 34/1035 (3.29%) neonates, Of babies born vaginally, 9/417 (2.16%) neonates tested positive for COVID-19 compared with 25/618 (4.05%) born by caesarean. |
| Neonatal deaths | Detailed neonatal outcomes and SARS-CoV-2 status were available for 1035 neonates born to mothers with confirmed COVID-19 infection. 618 neonates were born through cesarean section and 417 through vaginal delivery. | |  | (68 observational studies) | ⨁⨁◯◯ LOW | A total of six neonatal deaths (including one set of twins) and nine stillbirths (including one set of twins) have been reported. Of babies born vaginally, 0/417 (0.00%) neonatal deaths were reported compared with 6/618 (0.97%) born by cesarean. |
| Maternal deaths | 1019 pregnant women gave birth at the time of reporting. 603 gave birth by cesarean section and 416 by vaginal delivery. | |  | (68 observational studies) | ⨁⨁◯◯ LOW | 14 maternal deaths have been reported. 11 of these women gave birth through cesarean section and one by vaginal delivery. Two women died in the second trimester before delivery. |
| ***The risk in the intervention group** (and its 95% confidence interval) is based on the assumed risk in the comparison group and the **relative effect** of the intervention (and its 95% CI). **CI:** Confidence interval | | | | | | |
| **GRADE Working Group grades of evidence** **High certainty:** We are very confident that the true effect lies close to that of the estimate of the effect **Moderate certainty:** We are moderately confident in the effect estimate: The true effect is likely to be close to the estimate of the effect, but there is a possibility that it is substantially different **Low certainty:** Our confidence in the effect estimate is limited: The true effect may be substantially different from the estimate of the effect **Very low certainty:** We have very little confidence in the effect estimate: The true effect is likely to be substantially different from the estimate of effect | | | | | | |

**Appendix S6: Results of the sensitivity analyses for the primary outcome and secondary outcomes.**

|  | **Positive RT-PCR tests in neonates only^*^** | | **Included elevated levels of IgM in neonates^**^** | | **Asymptomatic women before delivery** | | **Symptomatic women before delivery** | | **Women delivered on first trimester** | | **Women delivered on second trimester** | | **Women delivered on third trimester** | |
| --- | --- | --- | --- | --- | --- | --- | --- | --- | --- | --- | --- | --- | --- | --- |
| **Outcomes** | V  No. (%)  (n=417) | CS  No. (%)  (n=615) | V  No. (%)  (n=417) | CS  No. (%)  (n=618) | V  No. (%)  (n=220) | CS  No. (%)  (n=174) | V  No. (%)  (n=196) | CS  No. (%)  (n=429) | V  No. (%)  (n=0) | CS  No. (%)  (n=0) | V  No. (%)  (n=3) | CS  No. (%)  (n=0) | V  No. (%)  (n=413) | CS  No. (%)  (n=603) |
| **SARS-CoV-2 test positivity in neonates**  **(primary outcome)** | 9  (2.16%) | 22 (3.58%) | 9 (2.16%) | 25 (4.05%) | NA | NA | NA | NA | NA | NA | NA | NA | NA | NA |
| **Neonatal deaths^***^**  **(secondary outcome)** | 0  (0%) | 6 (0.98%) | 0  (0%) | 6 (0.98%) | NA | NA | NA | NA | NA | NA | NA | NA | NA | NA |
| **Maternal deaths^****^**  **(secondary outcome)** | NA | NA | NA | NA | 0  (0%) | 2  (1.15%) | 1  (0.51%) | 9  (2.10%) | NA | NA | 0  (0%) | NA | 1  (0.24%) | 11  (1.82%) |

SARS-CoV-2 = severe acute respiratory syndrome coronavirus 2; V = vaginal delivery; CS = cesarean section; NA = not applicable.

**^*^** Including 21 positive SARS-CoV-2 RT-PCR test.

**^**^**Including 21 positive SARS-CoV-2 reverse transcriptase–polymerase chain reaction (RT-PCR) test and 3 (All three were born through cesarean section) elevated levels of ImmunoglobulinM (IgM) for SARS-CoV-2.

**^***^**Excluding 9 stillbirths.

**^****^**A total of fourteen maternal deaths have been reported, including two women died on second trimester before delivery.

**Appendix S7: Excluded studies**

**Reason for exclusion**: **Unpublished reports or not peer-reviewed (n=1)**

Liu, W.; Wang, Q.; Zhang, Q.; Chen, L.; Chen, J.; Zhang, B.; Lu, Y.; Wang, S.; Xia, L.; Huang, L.; Wang, K.; Liang, L.; Zhang, Y.; Turtle, L.; Lissauer, D.; Lan, K.; Feng, L.; Yu, H.; Liu, Y.; Sun, Z. Coronavirus Disease 2019 (COVID-19) During Pregnancy: A Case Series. Preprints 2020, 2020020373.

**Reason for exclusion**: **Unreported neonatal outcomes (n=3)**

Iqbal SN, Overcash R, Mokhtari N, et al. An Uncomplicated Delivery in a Patient with Covid-19 in the United States. N Engl J Med. 2020;382(16):e34.

He S, Wang D, Chi R, et al. A critically ill mother with COVID-19 in late pregnancy: a case report. Chin J Perinat Med, 2020,23(04): 220-223. DOI: 10.3760/cma.j.cn113903-20200228-00171.

Yu N, Li W, Kang Q, Zeng W, Feng L, Wu J. No SARS-CoV-2 detected in amniotic fluid in mid-pregnancy. Lancet Infect Dis. 2020.

**Reason for exclusion**: **Suspected case with COVID-19 (n=1)**

Yang H, Hu B, Zhan S, Yang LY, Xiong G. Effects of SARS-CoV-2 infection on pregnant women and their infants: A retrospective study in Wuhan, China. Archives of pathology & laboratory medicine. 2020.

**Reason for exclusion**: **Withdrawn (n=1)**

Karami P, Naghavi M, Feyzi A, et al. WITHDRAWN: Mortality of a pregnant patient diagnosed with COVID-19: A case report with clinical, radiological, and histopathological findings. Travel medicine and infectious disease. 2020:101665.

**Reason for exclusion**: **Duplicates (n=21)**

| Title | Setting | Time periods of recruitment (2020) | Language of publication | Study analysed (reference number) |
| --- | --- | --- | --- | --- |
| Clinical characteristics and intrauterine vertical transmission potential of COVID-19 infection in nine pregnant women:a retrospective review of medical records | Zhongnan Hospital | 1.20-1.31 | English | 45 |
| Perinatal novel coronavirus infection: a case report围产期新型冠状病毒感染一例 | Zhongnan Hospital | 1.20-2.03 | Chinese | 45 |
| Clinical characteristics and risk assessment of newborns born to mothers with COVID-19 | Zhongnan Hospital | 1.20-1.29 | English | 45 |
| Analysis of Vaginal Delivery Outcomes Among Pregnant Women in Wuhan, China During the COVID-19 Pandemic | Zhongnan Hospital | 1.20-3.02 | English | 45 |
| Analysis of the pregnancy outcomes in pregnant women with COVID-19 in Hubei Province湖北地区新型冠状病毒肺炎流行期间孕妇的妊娠结局分析 | Renmin Hospital | 1.30-2.17 | Chinese | 5 and 53 |
| Perinatal Transmission of COVID-19 Associated SARS-CoV-2: Should We Worry? | Renmin Hospital | 1.10-2.17 | English | 5 and 53 |
| Severe acute respiratory syndrome coronavirus 2(SARS-CoV-2) infection during late pregnancy: a report of 18 patients from Wuhan, China | Renmin Hospital | 1.30-3.01 | English | 5 and 53 |
| A case report of neonatal COVID-19 infection in China | Tongji Hospital | 2.01-2.18 | English | 14 and 28 |
| Severe Acute Respiratory Syndrome Coronavirus 2 (SARS-CoV-2) Vertical Transmission in Neonates Born to Mothers With Coronavirus Disease 2019 (COVID-19) Pneumonia | Tongji Hospital | 1.20-2.20 | English | 14 and 28 |
| Novel coronavirus pneumonia in pregnancy: perinatal outcomes妊娠晚期合并新冠肺炎的围生期结局 | Tongji Hospital | 1.01-2.12 | Chinese | 14 and 28 |
| Radiological findings and clinical characteristics of pregnant women with COVID-19 pneumonia | Central Hospital of Wuhan | 1.01-3.7 | English | 65 |
| Coronavirus disease 2019 in pregnancy | Central Hospital of Wuhan | 1.15-3.15 | English | 65 |
| Chest CT Findings in a Pregnant Patient with 2019 Novel Coronavirus Disease | Chongqing University Three Gorges Hospital | 2.09-2.11 | English | 23 |
| Novel Coronavirus Infection in Newborn Babies Under 28 Days in China | Data from the central government and local health departments | 1.01-3.13 | English | Not applicable |
| Coronavirus disease 2019 in pregnant women: a report based on 116 cases | Twenty-five hospitals in China | 1.20-3.24 | English | Not applicable |
| Clinical manifestations and outcome of SARS-CoV-2 infection during pregnancy | Hospital in areas outside Wuhan | 1.01-2.25 | English | Not applicable |
| Clinical Characteristics of Pregnant Women with Covid-19 in Wuhan, China | Data from the reporting system of the National Health Commission of China | 1.01-3.20 | English | Not applicable |
| Care of Critically Ill Pregnant Patients With Coronavirus Disease 2019: A Case Series | Maternal Child Health Research Center;  Hospital of the University of Pennsylvania | Not mention | English | 43 |
| Multidisciplinary Team Management and Cesarean Delivery for a Jordanian Woman Infected With SARS-COV-2: A Case Report | Jordan University of Science and Technology | Not mention | English | 26 |
| COVID-19 in pregnancy: early lessons | Columbia University Irving Medical Center | Not mention | English | 24 |
| SARS-CoV-2 infection in very preterm pregnancy: Experiences from two cases | Royal Berkshire Hospital | Not mention | English | 66 |

**Appendix S8: References for the Supplementary material**

1. Zhu H, Wang L, Fang C, et al. Clinical analysis of 10 neonates born to mothers with 2019-nCoV pneumonia. *Transl Pediatr* 2020; **9**(1): 51-60.

2. Li M, XU M, Zhan W, Han T, Zhang G, Lu Y. Report of the first cases of mother and infant infections with 2019 novel coronavirus in Xinyang City Henan Province *Chin J Infect Dis* 2020; **38**.

3. Wang X, Zhou Z, Zhang J, Zhu F, Tang Y, Shen X. A case of 2019 Novel Coronavirus in a pregnant woman with preterm delivery. *Clin Infect Dis* 2020.

4. Chen S, Huang B, Luo D, et al. Pregnancy with new coronavirus infection: clinical characteristics and placental pathological analysis of three case. *Chin J Pathol* 2020; **49**(02): 418-23.

5. Lei D, Wang C, Li C, et al. Clinical characteristics of COVID-19 in pregnancy: analysis of nine cases. *Chin J Perinat Med* 2020; **23**(03): 159-65.

6. Yao L, Wang J, Zhao J, Cui J, Hu Z. Asymptomatic COVID-19 infection in pregnant woman in the third trimester: a case report. *Chin J Perinat Med* 2020; **23**.

7. Zhao Y, Lin Z, Mao X, Jiang X, Zhang W. Dyspnea in a newborn infant born to mother suffering from novel coronavirus pneumonia. *Chin J Neonatol* 2020; **35**(02): 84-5.

8. Bai B, Gu Z, Hu S, et al. Pathogen survey in a mother with novel coronavirus pneumonia and her newborn infant. *Chin J Neonatol* 2020; **35**(02): 85-6.

9. Kang X, Zhang R, He H, et al. [Anesthesia management in cesarean section for a patient with coronavirus disease 2019]. Zhejiang da xue xue bao Yi xue ban = Journal of Zhejiang University Medical sciences 2020; 49(1): 249-52.

10. Chen Y, Peng H, Wang L, et al. Infants Born to Mothers With a New Coronavirus (COVID-19). *Frontiers in pediatrics* 2020; **8**: 104.

11. Zhou R, Chen Y, Lin C, et al. Asymptomatic COVID-19 in pregnant woman with typical chest CT manifestation: a case report. *Chin J Perinat Med* 2020; **23**(03): 166-8.

12. Khan S, Peng L, Siddique R, et al. Impact of COVID-19 infection on pregnancy outcomes and the risk of maternal-to-neonatal intrapartum transmission of COVID-19 during natural birth. *Infection control and hospital epidemiology* 2020; **41**(6): 748-50.

13. Li N, Han L, Peng M, et al. Maternal and neonatal outcomes of pregnant women with COVID-19 pneumonia: a case-control study. *Clin Infect Dis* 2020.

14. Yu N, Li W, Kang Q, et al. Clinical features and obstetric and neonatal outcomes of pregnant patients with COVID-19 in Wuhan, China: a retrospective, single-centre, descriptive study. *Lancet Infect Dis* 2020.

15. Zeng L, Xia S, Yuan W, et al. Neonatal Early-Onset Infection With SARS-CoV-2 in 33 Neonates Born to Mothers With COVID-19 in Wuhan, China. *JAMA Pediatr* 2020.

16. Zeng H, Xu C, Fan J, et al. Antibodies in Infants Born to Mothers With COVID-19 Pneumonia. JAMA 2020.

17. Dong L, Tian J, He S, et al. Possible Vertical Transmission of SARS-CoV-2 From an Infected Mother to Her Newborn. *JAMA* 2020.

18. Chen S, Liao E, Cao D, Gao Y, Sun G, Shao Y. Clinical analysis of pregnant women with 2019 novel coronavirus pneumonia. *J Med Virol* 2020.

19. Baud D, Greub G, Favre G, et al. Second-Trimester Miscarriage in a Pregnant Woman With SARS-CoV-2 Infection. *Jama* 2020; **323**(21): 2198-200.

20. Lee DH, Lee J, Kim E, Woo K, Park HY, An J. Emergency cesarean section on severe acute respiratory syndrome coronavirus 2 (SARS- CoV-2) confirmed patient. *Korean journal of anesthesiology* 2020.

21. Kalafat E, Yaprak E, Cinar G, et al. Lung ultrasound and computed tomographic findings in pregnant woman with COVID-19. Ultrasound in obstetrics & gynecology : the official journal of the International Society of Ultrasound in Obstetrics and Gynecology 2020; 55(6): 835-7.

22. Gidlöf S, Savchenko J, Brune T, Josefsson H. COVID-19 in pregnancy with comorbidities: More liberal testing strategy is needed. *Acta obstetricia et gynecologica Scandinavica* 2020; **99**(7): 948-9.

23. Peng Z, Wang J, Mo Y, et al. Unlikely SARS-CoV-2 vertical transmission from mother to child: A case report. *Journal of infection and public health* 2020; **13**(5): 818-20.

24. Breslin N, Baptiste C, Gyamfi-Bannerman C, et al. COVID-19 infection among asymptomatic and symptomatic pregnant women: Two weeks of confirmed presentations to an affiliated pair of New York City hospitals. *American journal of obstetrics & gynecology MFM* 2020; **2**(2): 100118.

25. Xiong X, Wei H, Zhang Z, et al. Vaginal delivery report of a healthy neonate born to a convalescent mother with COVID--19. *J Med Virol* 2020.

26. Khassawneh M, Khasawneh W, Zaghal LA, Hayajneh W, Abdelal F. The first Jordanian newborn delivered to COVID-19 infected mother with no evidence of vertical transmission: A case report. *Research Square* 2020.

27. Schnettler WT, Al Ahwel Y, Suhag A. Severe acute respiratory distress syndrome in coronavirus disease 2019-infected pregnancy: obstetric and intensive care considerations. American journal of obstetrics & gynecology MFM 2020; 2(3): 100120.

28. Liu W, Wang J, Li W, Zhou Z, Liu S, Rong Z. Clinical characteristics of 19 neonates born to mothers with COVID-19. *Frontiers of medicine* 2020; **14**(2): 193-8.

29. Carosso A, Cosma S, Borella F, et al. Pre-labor anorectal swab for SARS-CoV-2 in COVID-19 pregnant patients: is it time to think about it? *European journal of obstetrics, gynecology, and reproductive biology* 2020; **249**: 98-9.

30. González Romero D, Ocampo Pérez J, González Bautista L, Santana-Cabrera L. [Pregnancy and perinatal outcome of a woman with COVID-19 infection]. Revista clinica espanola. 2020;220(8):533-534.

31. Vlachodimitropoulou Koumoutsea E, Vivanti AJ, Shehata N, et al. COVID-19 and acute coagulopathy in pregnancy. Journal of thrombosis and haemostasis : JTH 2020; 18(7): 1648-52.

32. Zamaniyan M, Ebadi A, Aghajanpoor S, Rahmani Z, Haghshenas M, Azizi S. Preterm Delivery in Pregnant Woman With Critical COVID-19 Pneumonia and Vertical Transmission. *Prenatal diagnosis* 2020; **n/a**(n/a).

33. Alzamora MC, Paredes T, Caceres D, Webb CM, Valdez LM, La Rosa M. Severe COVID-19 during Pregnancy and Possible Vertical Transmission. *American journal of perinatology* 2020; **37**(8): 861-5.

34. Lyra J, Valente R, Rosário M, Guimarães M. Cesarean Section in a Pregnant Woman with COVID-19: First Case in Portugal. *Acta medica portuguesa* 2020; **33**(6): 429-31.

35. Al-kuraishy H, Al-Maiahy T, Al-Gareeb A, Musa R, Ali Z. COVID-19 pneumonia in an Iraqi pregnant woman with preterm delivery. *Asian Pacific Journal of Reproduction* 2020; **9**(3): 156-8.

36. Lu D, Sang L, Du S, Li T, Chang Y, Yang XA. Asymptomatic COVID-19 infection in late pregnancy indicated no vertical transmission. *J Med Virol* 2020.

37. Ferrazzi E, Frigerio L, Savasi V, et al. Vaginal delivery in SARS-CoV-2-infected pregnant women in Northern Italy: a retrospective analysis. *BJOG : an international journal of obstetrics and gynaecology* 2020.

38. Hantoushzadeh S, Shamshirsaz AA, Aleyasin A, et al. Maternal death due to COVID-19. *Am J Obstet Gynecol* 2020; **223**(1): 109.e1-.e16.

39. Penfield CA, Brubaker SG, Limaye MA, et al. Detection of SARS-COV-2 in Placental and Fetal Membrane Samples. *American journal of obstetrics & gynecology MFM* 2020: 100133.

40. Wu Y, Liu C, Dong L, et al. Coronavirus disease 2019 among pregnant Chinese women: case series data on the safety of vaginal birth and breastfeeding. *BJOG : an international journal of obstetrics and gynaecology* 2020.

41. Piersigilli F, Carkeek K, Hocq C, et al. COVID-19 in a 26-week preterm neonate. The Lancet Child & adolescent health 2020; 4(6): 476-8.

42. Blauvelt CA, Chiu C, Donovan AL, et al. Acute Respiratory Distress Syndrome in a Preterm Pregnant Patient With Coronavirus Disease 2019 (COVID-19). *Obstetrics and gynecology* 2020.

43. Pierce-Williams RAM, Burd J, Felder L, et al. Clinical course of severe and critical COVID-19 in hospitalized pregnancies: a US cohort study. *American journal of obstetrics & gynecology MFM* 2020: 100134.

44. Polónia-Valente R, Moucho M, Tavares M, Vilan A, Montenegro N, Rodrigues T. Vaginal delivery in a woman infected with SARS-CoV-2 - The first case reported in Portugal. *European journal of obstetrics, gynecology, and reproductive biology* 2020; **250**: 253-4.

45. Liu P, Zheng J, Yang P, et al. The immunologic status of newborns born to SARS-CoV-2-infected mothers in Wuhan, China. *The Journal of allergy and clinical immunology* 2020.

46. Perrone S, Deolmi M, Giordano M, et al. Report of a series of healthy term newborns from convalescent mothers with COVID-19. *Acta bio-medica : Atenei Parmensis* 2020; **91**(2): 251-5.

47. Baergen RN, Heller DS. Placental Pathology in Covid-19 Positive Mothers: Preliminary Findings. *Pediatric and developmental pathology : the official journal of the Society for Pediatric Pathology and the Paediatric Pathology Society* 2020; **23**(3): 177-80.

48. Taghizadieh A, Mikaeili H, Ahmadi M, Valizadeh H. Acute kidney injury in pregnant women following SARS-CoV-2 infection: A case report from Iran. Respiratory medicine case reports 2020; 30: 101090.

49. Patanè L, Morotti D, Giunta MR, et al. Vertical transmission of COVID-19: SARS-CoV-2 RNA on the fetal side of the placenta in pregnancies with COVID-19 positive mothers and neonates at birth. *American journal of obstetrics & gynecology MFM* 2020: 100145.

50. Kirtsman M, Diambomba Y, Poutanen SM, et al. Probable congenital SARS-CoV-2 infection in a neonate born to a woman with active SARS-CoV-2 infection. *CMAJ : Canadian Medical Association journal = journal de l'Association medicale canadienne* 2020; **192**(24): E647-e50.

51. Dória M, Peixinho C, Laranjo M, Mesquita Varejão A, Silva PT. Covid-19 during pregnancy: A case series from an universally tested population from the north of Portugal. European journal of obstetrics, gynecology, and reproductive biology 2020; 250: 261-2.

52. Mehta H, Ivanovic S, Cronin A, et al. Novel coronavirus-related acute respiratory distress syndrome in a patient with twin pregnancy: A case report. *Case reports in women's health* 2020; **27**: e00220.

53. Chen R, Zhang Y, Huang L, Cheng BH, Xia ZY, Meng QT. Safety and efficacy of different anesthetic regimens for parturients with COVID-19 undergoing Cesarean delivery: a case series of 17 patients. *Canadian journal of anaesthesia = Journal canadien d'anesthesie* 2020; **67**(6): 655-63.

54. Sharma KA, Kumari R, Kachhawa G, et al. Management of the first patient with confirmed COVID-19 in pregnancy in India: From guidelines to frontlines. *International journal of gynaecology and obstetrics: the official organ of the International Federation of Gynaecology and Obstetrics* 2020; **150**(1): 116-8.

55. Xia H, Zhao S, Wu Z, Luo H, Zhou C, Chen X. Emergency Caesarean delivery in a patient with confirmed COVID-19 under spinal anaesthesia. *British journal of anaesthesia* 2020; **124**(5): e216-e8.

56. Panichaya P, Thaweerat W, Uthaisan J. Prolonged viral persistence in COVID-19 second trimester pregnant patient. European journal of obstetrics, gynecology, and reproductive biology 2020; 250: 263.

57. Lokken EM, Walker CL, Delaney S, et al. Clinical Characteristics of 46 Pregnant Women with a SARS-CoV-2 Infection in Washington State. *Am J Obstet Gynecol* 2020.

58. London V, McLaren R, Jr., Atallah F, et al. The Relationship between Status at Presentation and Outcomes among Pregnant Women with COVID-19. *American journal of perinatology* 2020.

59. Li Y, Zhao R, Zheng S, et al. Lack of Vertical Transmission of Severe Acute Respiratory Syndrome Coronavirus 2, China. *Emerg Infect Dis* 2020; **26**(6): 1335-6.

60. Qadri F, Mariona F. Pregnancy affected by SARS-CoV-2 infection: a flash report from Michigan. *The journal of maternal-fetal & neonatal medicine : the official journal of the European Association of Perinatal Medicine, the Federation of Asia and Oceania Perinatal Societies, the International Society of Perinatal Obstet* 2020: 1-3.

61. Tang MW, Nur E, Biemond BJ. Immune thrombocytopenia due to COVID-19 during pregnancy. American Journal of Hematology 2020; 95(8): E191-E2.

62. Lowe B, Bopp B. COVID-19 vaginal delivery - A case report. *The Australian & New Zealand journal of obstetrics & gynaecology* 2020; **60**(3): 465-6.

63. Kayem G, Lecarpentier E, Deruelle P, et al. A snapshot of the Covid-19 pandemic among pregnant women in France. *Journal of gynecology obstetrics and human reproduction* 2020: 101826.

64. Martínez-Perez O, Vouga M, Cruz Melguizo S, et al. Association Between Mode of Delivery Among Pregnant Women With COVID-19 and Maternal and Neonatal Outcomes in Spain. *Jama* 2020.

65. Wang Z, Wang Z, Xiong G. Clinical characteristics and laboratory results of pregnant women with COVID-19 in Wuhan, China. *International journal of gynaecology and obstetrics: the official organ of the International Federation of Gynaecology and Obstetrics* 2020.

66. Knight M, Bunch K, Vousden N, et al. Characteristics and outcomes of pregnant women admitted to hospital with confirmed SARS-CoV-2 infection in UK: national population based cohort study. *BMJ (Clinical research ed)* 2020; **369**: m2107.

67. Pereira A, Cruz-Melguizo S, Adrien M, Fuentes L, Marin E, Perez-Medina T. Clinical course of coronavirus disease-2019 in pregnancy. *Acta obstetricia et gynecologica Scandinavica* 2020; **99**(7): 839-47.

68. Bani Hani DA, Alsharaydeh I, Bataineh AM, et al. Successful Anesthetic Management in Cesarean Section for Pregnant Woman with COVID-19. *The American journal of case reports* 2020; **21**: e925512.
